# Correlation of the NBME Advanced Clinical Examination in EM and the National EM M4 exams

**DOI:** 10.5811/westjem.2014.11.24189

**Published:** 2015-01-05

**Authors:** Katherine Hiller, Emily S. Miller, Luan Lawson, David Wald, Michael Beeson, Corey Heitz, Thomas Morrissey, Joseph House, Stacey Poznanski

**Affiliations:** * University of Arizona, Department of Emergency Medicine, Tucson, Arizona; † Harvard University, Department of Emergency Medicine, Boston, Massachusetts; ‡ Brody School of Medicine at East Carolina University, Department of Emergency Medicine, Greenville, North Carolina; § Temple University School of Medicine, Department of Emergency Medicine, Philadelphia, Pennsylvania; ¶ Northeastern Ohio Medical University, Department of Emergency Medicine, Rootstown, Ohio; || Virginia Tech Carilion School of Medicine, Department of Emergency Medicine, Roanoke, Virginia; # University of Florida Health Sciences Center, Department of Emergency Medicine, Jacksonville, Florida; ** University of Michigan School of Medicine, Department of Emergency Medicine, Ann Arbor, Michigan; †† Wright State University Boonshoft School of Medicine, Department of Emergency Medicine, Dayton, Ohio

## Abstract

**Introduction:**

Since 2011 two online, validated exams for fourth-year emergency medicine (EM) students have been available (National EM M4 Exams). In 2013 the National Board of Medical Examiners offered the Advanced Clinical Examination in Emergency Medicine (EM-ACE). All of these exams are now in widespread use; however, there are no data on how they correlate. This study evaluated the correlation between the EM-ACE exam and the National EM M4 Exams.

**Methods:**

From May 2013 to April 2014 the EM-ACE and one version of the EM M4 exam were administered sequentially to fourth-year EM students at five U.S. medical schools. Data collected included institution, gross and scaled scores and version of the EM M4 exam. We performed Pearson’s correlation and random effects linear regression.

**Results:**

303 students took the EM-ACE and versions 1 (V1) or 2 (V2) of the EM M4 exams (279 and 24, respectively). The mean percent correct for the exams were as follows: EM-ACE 74.8 (SD-8.83), V1 83.0 (SD-6.41), V2 78.5 (SD-7.70). Pearson’s correlation coefficient for the V1/EM-ACE was 0.51 (0.42 scaled) and for the V2/EM-ACE was 0.59 (0.41 scaled). The coefficient of determination for V1/EM-ACE was 0.72 and for V2/EM-ACE = 0.71 (0.86 and 0.49 for scaled scores). The R-squared values were 0.25 and 0.30 (0.18 and 0.13, scaled), respectively. There was significant cluster effect by institution.

**Conclusion:**

There was moderate positive correlation of student scores on the EM-ACE exam and the National EM M4 Exams.

## INTRODUCTION

Clerkship directors employ numerous methods to assess medical student performance during clinical rotations. These methods include direct observation, clinical feedback from supervisors, performance on written and/or oral examinations, simulation, and case presentations. A 2010 survey of emergency medicine (EM) clerkship directors revealed that the most commonly used methods in determining a medical student’s clerkship grade are clinical performance assessment forms (used by 94% of clerkships) and written exams (used by 57% of clerkships).^1^ On average, written exam scores count for 24.5% of students’ grades on an EM clerkship.^1^ Unlike most other core clerkships (Surgery, Pediatrics, Obstetrics/Gynecology and Internal Medicine), until recently, EM has not had a nationally accepted, standardized exam such as the National Board of Medical Examiners “shelf exam,” or Advanced Clinical Exam in Emergency Medicine (NBME EM-ACE). Historically, clerkship directors relied on creating their own exams for an end-of-rotation written assessment of medical knowledge. Most clerkship directors have no formal training in exam item writing, making the level of quality of these internal exams difficult to ascertain. Further, until 2006, when a standardized national fourth-year curriculum was published, there had been significant variability in the “core content” material for rotations at different venues.^2^ This curriculum was updated in 2010.^3^

With a new national curriculum in place, the Clerkship Directors of Emergency Medicine (CDEM) released the first version (V1) of a national, standardized, end-of-rotation exam for fourth-year students in 2011 entitled the National EM M4 Exam (referred to by some as the CDEM Exam or Society for Academic Emergency Medicine tests exam).^4^,^5^ This exam was created to assess content in the published EM curriculum and consists of items written according to published item-writing guidelines.^5^ CDEM released a second version (V2) of the National EM M4 Exam in 2012.^6^ Both versions of the National EM M4 Exam are available online free of charge to all U.S. clerkship directors (www.saemtests.org).

In 2013, the NBME introduced the Emergency Medicine Advanced Clinical Exam (EM-ACE), which was written and developed by an NBME task force consisting of CDEM members with formal training in item writing. The EM-ACE was made available free of charge from its initial release in April 2013 until June 2014. Like the National EM M4 Exams, the NBME EM-ACE is based on content in the published fourth-year EM curriculum, making the curricula covered theoretically identical.^2^,^3^

Before a stable national curriculum was agreed upon, it was not possible to generate a standardized end-of-rotation assessment tool for EM students, and comparison of student performance across institutions was not feasible. The release of these end-of-rotation examinations represents the first opportunity for EM clerkship directors to be able to assess their students with a standardized, nationally available assessment tool. Although both exams are based on the same national curriculum, it is unknown whether student performance on the NBME EM-ACE correlates with performance on the National EM M4 Exams. End-of-rotation exam scores are typically included in a student’s summative grade report and may also be included in letters of evaluation for residency application. Understanding how scores on the NBME EM-ACE correlate with scores on the National EM M4 Exams would help inform educators and program directors about individual students and more importantly, enable comparison of students who have taken different exams. The objective of this study was to correlate medical student performance on the NBME EM-ACE with medical student performance on V1 and V2 of the National EM M4 Exams.

## METHODS

This multicenter, prospective, paired comparison study was performed across five U.S. allopathic medical schools from May 2014 to April 2014. All fourth-year medical students participating in a fourth-year EM rotation at the study sites were administered both the NBME EM-ACE and an EM M4 exam. The study sites varied with regard to having mandatory, selective or elective EM rotations, but were all four weeks long and used the standardized curriculum recommended by CDEM. Study sites administered either V1 or V2 of the EM M4 exam based upon site preference. Exams were taken consecutively within one day of each other, at the end of the rotation. Individual study sites determined which exam was administered first. Both exams were administered by the same clerkship coordinator or other administrator according to respective protocols developed by the NBME and CDEM. At all sites, students were aware that the EM M4 exam would count towards their grade, as per local institution protocol. Without longitudinal performance data or norms, most sites did not count NBME exam towards the final rotation grade; however, to encourage students to take the NBME exam seriously, some institutions advised students that although the NBME exam could not lower their grade, a strong performance would be reflected in their final evaluation. One institution used the NBME score for a small portion (5%) of the final course grade.

The clerkship director or coordinator collected deidentified data, which included institution, NBME gross score (percent correct, when available), NBME scaled score, the version of the EM M4 exam administered (V1 and V2) and the gross score on that exam. We pooled the data and calculated Pearson correlation coefficients for the NBME (gross and scaled) and EM M4 (V1 and V2) exam scores. Random effects linear regression with institution as the cluster variable was performed for both EM M4 versions.

We performed data collection in Microsoft Excel 2007. Data analysis was performed with StataMP 11.0 (College Station, TX).

This project was determined to be exempt from human subjects review by the University of Arizona Institutional Review Board.

## RESULTS

**Figure 1:**
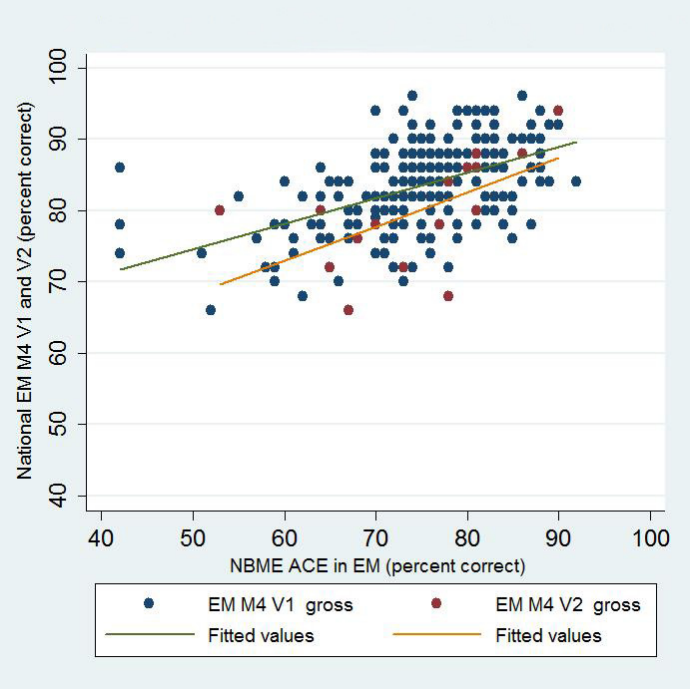
Correlation of NBME EM-ACE and National EM M4 V1 and V2. *EM,* emergency medicine; *V1,* first version; V2, second version; *NBME,* National Board of Medical Examiners; *EM-ACE,* Advanced Clinical Exam in Emergency Medicine

**Figure 2:**
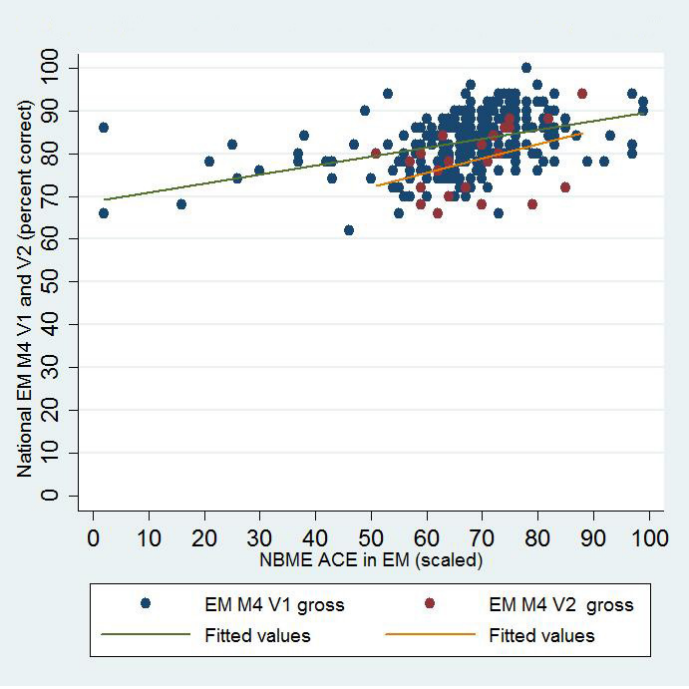
Correlation of NBME EM-ACE and National EM M4 V1 and V2. *EM,* emergency medicine; *V1,* first version; V2, second version; *NBME,* National Board of Medical Examiners; *EM-ACE,* Advanced Clinical Exam in Emergency Medicine

Five institutions administered both the NBME EM-ACE and one version of the EM M4 exam to 303 fourth-year students at the end of their EM rotation. V1 of the EM M4 was administered to 279 students, and V2 to 24 students. This profile is similar to the national distribution of students who took V1 and V2 in 2013–14 (5060 and 787, respectively).^7^ The mean NBME raw score was 74.8 (n=216; SD 8.83). The mean NBME scaled score for the entire cohort was 68.2 (SD 12.8). The mean EM M4 V1 raw score was 83.0 (n=279; SD 6.41), and the mean EM M4 V2 raw score was 78.5 (n=24, SD 7.80). We performed Pearson’s correlations and linear regression on the NBME raw and scaled scores and V1 and V2 of the EM M4 exams. There was moderate positive correlation for all comparisons (Figures 1 and 2). There was a cluster effect for institution, so it was retained in the linear regression analysis for both the EM M4 versions (Table 1).

## DISCUSSION

The availability of the NBME EM-ACE this past year is of great importance to our specialty. The NBME has provided internal validity data for the EM-ACE. However, the impact the NBME EM-ACE will likely have necessitates assessment of external validity and reliability compared to what historically has been used as the assessment standard. This study represents the first step in this evolving process.

End-of-rotation exams play a high stakes role in medical student evaluations. Although they are imperfect tools in that they provide only partial assessment of a student’s level of competence (namely, medical knowledge and problem solving), they remain one of few objective quantifiable tools available to medical student educators for assessment of a student’s performance. A 2009 survey revealed that 88% of U.S. and Canadian internal medicine clerkships administer the NBME subject exam in medicine.^8^ Final exam scores are often reported in a student’s summative evaluation.

EM differs from other core clerkships in its position and timing in the medical school curriculum; not all schools require an EM rotation, and most EM rotations occur in the fourth year.^1^ A 2014 survey of EM clerkship directors revealed that EM is a required rotation at 52% of medical schools.^1^ This percentage has risen from a similar survey in 2007, in which EM was a required rotation at only 36% of medical schools.^9^ As medical schools increasingly adopt EM as a core clerkship, the need for standardized end-of-rotation exam options will likely continue to rise.

Many EM rotations are completed away from a student’s home institution, either because the home institution lacks a robust academic EM training program, or because the student chooses externships as audition rotations in preparation for entering the residency match. Scores from end-of-rotation exams are not only reported in end-of-rotation summative evaluations, but also frequently reflected in letters of recommendation from these externships. With EM being a popular specialty choice and competition for EM residency positions increasing, any objective measure of student performance has the potential to have a profound effect on a student’s candidacy for residency.^10^ Correlation and comparison data between the NBME and EM M4 exams provides the ability to compare applicants who have taken different exams.

Importantly, cost is also a factor. As more medical schools require students to complete an EM clerkship, there may be increased use of the NBME EM-ACE. The exam was provided free to clerkships for the first year. Starting in July 2014, there has been a $41 per student fee for use of this exam.^11^ Some EM clerkships may have funding through their medical school or department to cover such expenses; however many EM clerkships likely do not have a readily available funding source for student exam fees. Schools may be even less likely to fund the exam for visiting externs. If the cost of the exam is deferred to students, this may limit a student’s ability to accept externships. The National EM M4 Exams have been offered free of charge since their release and would remain a viable option for clerkship directors without access to funding for the NBME EM-ACE. The usage of the National EM M4 Exams is likely to remain common. It is notable that nationwide usage of the National EM M4 Exams has remained steady since the release of the NBME EM-ACE, reflecting a continued need for these exams.^7^ Given the high likelihood that both the NBME EM-ACE and the National EM M4 Exams will continue to be used to assess EM students, the ability to compare student performance on these exams is advantageous.

While it is not surprising that exams based on the same core curriculum and written by trained item writers would yield a positive correlation, the documentation of this correlation is helpful for the reasons discussed above. The observed positive correlation between student performance on the National EM M4 Exams and the NBME EM-ACE is also encouraging because it suggests that the National EM M4 Exams, which were created on a limited budget by national EM educators who volunteered their time and efforts, are able to effectively assess medical student knowledge comparably to the EM exam offered by the NBME, which is considered the gold standard for student exams.

## LIMITATIONS

The first limitation to acknowledge is the validity of multiple-choice questions as a tool in the assessment of student performance. While multiple-choice written exam questions may only provide a partial assessment of medical knowledge and perhaps basic clinical reasoning skills, they are a routine part of assessment at virtually every level of training from grade school and high school, through college, medical school, residency, and the board certification and recertification processes. Even as newer assessment techniques, such as simulation and online interactive cases, continue to be developed, the multiple-choice question remains a frequently used assessment tool and piece of a student’s overall assessment.

Another possible limitation of this study is that all of the authors were involved in the development of the National EM M4 Exams, and several were involved in the development of the NBME EM-ACE (KH, EM, LL, DW, CH). Theoretically, this could bias the results towards a positive correlation. It is unlikely that this bias, if present, altered the results of the study, as the population who took the test was heterogenous, geographically diverse, and the conclusion robust. Additionally, though the same item-writers were working with the same curriculum and core content, the items themselves were vetted and edited by an outside organization (NBME) and the process of item writing itself was significantly different in the two systems.

An additional limitation of this study is that the data provided by the NBME came in the form of raw data (percent correct) for the first six months of the study, but was not available as such for the last half of the study. Scaled scores were available retrospectively for the first half of the study and from October until completion of data collection. We chose to report both the raw and scaled score correlations, however, the NBME will only be reporting scaled scores going forward. As noted above, correlation existed for both raw score and scaled score data.

The number of students completing V2 was low (n=24) compared the number of students completing V1 (n=279). This ratio is in line with the ratio of V1 and V2 exams that have been completed since inception, 5,060 and 787, respectively.^7^ Future studies could obtain more data for V2 examinees.

Another potential limitation to our study is that the number of EM rotations completed by a student was not collected as a potential confounding variable. Many students, especially those applying in EM, complete more than one EM rotation. A more experienced student could be expected to perform better on an end-of-rotation exam than a student who has completed only one EM rotation. It is unlikely that greater EM experience would introduce a systematic bias when comparing exam performance on two exams by the same student, however. This represents an area for future study.

One last potential limitation is how students’ scores were used, i.e. students’ grades were derived almost exclusively from their scores on the National EM M4 Exams, while performance on the NBME EM-ACE was not “high stakes.” Students may have been more motivated to score well on the EM M4 exam as compared to the NBME exam, which could have resulted in lower scores on this exam.

## CONCLUSION

Two standardized, end-of-rotation exam options for fourth-year EM students currently exist, the National EM M4 Exams and the NBME EM-ACE. There is a modest positive correlation in student performance on these exams, suggesting that both exams are effective in assessing EM student knowledge of the published fourth-year EM student curriculum, and enabling comparison of student performance between students completing different exams. While this correlation does not completely address whether either exam is effective at assessing students’ comprehension of the EM core curriculum, it is encouraging and does suggest that either exam is effective as an end of rotation assessment method.

## Figures and Tables

**Table 1:** Pearson’s correlation coefficient and R-squared values for the NBME EM-ACE (raw and scaled scores) and V1 and V2 of the EM M4 exam.

Comparison	Pearson’s Correlation coefficient	R-squared value
NBME (raw) version V1 EM M4	0.51	0.26
NBME (scaled) version V1 EM M4	0.42	0.18
NBME (raw) version V2 EM M4	0.58	0.30
NBME (scaled) version V2 EM M4	0.41	0.13

*NBME,* National Board of Medical Examiners; *EM-ACE,* Advanced Clinical Exam in Emergency Medicine; *EM,* emergency medicine; *V1,* first version; V2, second version
