# This article corrects: “Correlation of the NBME Advanced Clinical Examination in EM and the National EM M4 exams”

**DOI:** 10.5811/westjem.2015.2.25829

**Published:** 2015-03-12

**Authors:** Katherine Hiller, Emily S. Miller, Luan Lawson, David Wald, Michael Beeson, Corey Heitz, Thomas Morrissey, Joseph House, Stacey Poznanski

**Affiliations:** * University of Arizona, Department of Emergency Medicine, Tucson, Arizona; † Harvard University, Department of Emergency Medicine, Boston, Massachusetts; ‡ Brody School of Medicine at East Carolina University, Department of Emergency Medicine, Greenville, North Carolina; § Temple University School of Medicine, Department of Emergency Medicine, Philadelphia, Pennsylvania; ¶ Northeastern Ohio Medical University, Department of Emergency Medicine, Rootstown, Ohio; || Virginia Tech Carilion School of Medicine, Department of Emergency Medicine, Roanoke, Virginia; # University of Florida Health Sciences Center, Department of Emergency Medicine, Jacksonville, Florida; ** University of Michigan School of Medicine, Department of Emergency Medicine, Ann Arbor, Michigan; †† Wright State University Boonshoft School of Medicine, Department of Emergency Medicine, Dayton, Ohio

In the Original Research article entitled “Correlation of the National Board of Medical Examiners Advanced Clinical Examination in Emergency Medicine and the National Emergency Medicine M4 Exams,” published in the January 2015 issue of the *Western Journal of Emergency Medicine* (2015;16(1):138–142. DOI: 10.5811/westjem.2014.11.24189), there were the following errors in the published article:

1. On page 138, the 1st line of the results section of the abstract should read: 305 students took the EM-ACE and versions 1 (V1) or 2 (V2) of the EM M4 exams (281 and 24, respectively).
2. On page 138, the 2nd line of the results section of the abstract should read: The mean percent correct for the exams were as follows: EM-ACE 74.9 (SD-9.82), V1 83.0 (SD-6.39), V2 78.5 (SD-7.70).
3. On page 138, the 3rd line of the results section of the abstract should read: Pearson’s correlation coefficient for the V1/EM-ACE was 0.53 (0.43 scaled) and for the V2/EM-ACE was 0.58 (0.41 scaled).
4. On page 138, the 4th line of the results section of the abstract should read: The coefficient of determination for V1/EM-ACE was 0.73 and for V2/EM-ACE 0.71 (0.65 and .49 for scaled scores) [ERRATUM]. The R-squared values were 0.28 and 0.30 (0.18 and 0.13 scaled), respectively.
5. On page 140, the 1st line of the results section should read: Five institutions administered both the NBME EM-ACE and one version of the EM M4 exam to 305 fourth-year students at the end of their EM rotation. V1 of the EM M4 was administered to 281 students, and V2 to 24 students.
6. On page 140, the fifth sentence of the results section should read: The mean NBME scaled score for the entire cohort was 68.3 (SD-9.66).
7. On page 141 in the table, the Pearson’s Correlation coefficient for “NBME (raw) V1 EM M4” should be 0.53 and “NBME (scaled) V1 EM M4” 0.43.
8. On page 141 in the table, the R-squared value for “NBME (raw) V1 EM M4” should be 0.28.
9. The correct Figures for “Figure 1” and Figure 2” on page 140 are shown on the following page:

We apologize for this error.

## Figures and Tables

**Figure 1:**
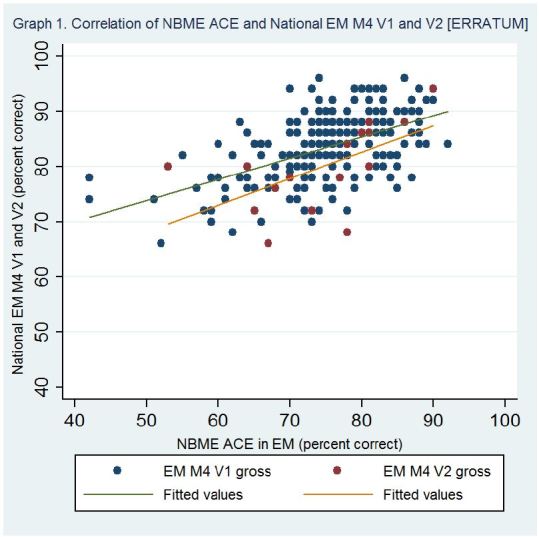
Correlation of NBME EM-ACE and National EM M4 V1 and V2. *EM,* emergency medicine; *V1,* first version; V2, second version; *NBME,* National Board of Medical Examiners; *EM-ACE,* Advanced Clinical Exam in Emergency Medicine

**Figure 2:**
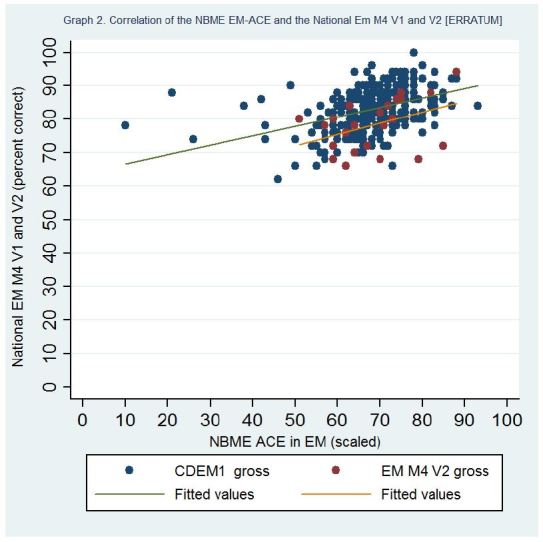
Correlation of NBME EM-ACE and National EM M4 V1 and V2. *EM,* emergency medicine; *V1,* first version; V2, second version; *NBME,* National Board of Medical Examiners; *EM-ACE,* Advanced Clinical Exam in Emergency Medicine
